# Integrated above- and below-ground interplant cueing of salt stress

**DOI:** 10.1080/15592324.2025.2542560

**Published:** 2025-08-12

**Authors:** Kai Ito, Haruna Ohsaki, Ariel Novoplansky, Shun K. Hirota, Akira Yamawo

**Affiliations:** a Department of Biology, Faculty of Agriculture and Life Science, Hirosaki University, Hirosaki, Japan; b Mitrani Department of Desert Ecology, Blaustein Institutes for Desert Research, Ben-Gurion University of the Negev, Beersheba, Israel; c Botanical Gardens, Osaka Metropolitan University, Osaka, Japan; d Center for Ecological Research, Kyoto University, Otsu, Japan

**Keywords:** Kin recognition, *Plantago asiatica*, plant communication, root exudates, stress cueing, volatile organic compounds

## Abstract

Neighboring plants exchange adaptive information related to their genetic identity, stress experiences, and reproductive state. Here, we tested the possibility that *Plantago asiatica* plants utilize both above- and below-ground communication to differentially respond to stress cues perceived from neighbors with variable genetic identities. Stress response was observed by recording stomatal aperture in stressed plants and their neighbors while restricting interplant communication to either root or shoot cueing. Split-root plants were planted in triplets at equidistant intervals. Half of the roots of the central plant were subjected to salt stress, while the other half shared its rooting volume with the roots of an unstressed neighboring plant on one side, and its headspace with another unstressed neighbor on the other side. Sixty minutes after the onset of salt stress, soil-sharing neighbors had a larger proportion of closed stomata when the stressed plant was genetically closer (sibling [SB] or from a near population [NP]) than from a more remote population (FP). In contrast, aboveground stress cueing was equally effective regardless of the genetic relatedness of the neighboring plants. The findings demonstrate for the first time a concurrent utilization of both specific and nonspecific interplant stress cueing. The results call for further investigation into the adaptive implications of these communication modes on the survival and performance of *P. asiatica* under variable environmental scenarios.

## Introduction

Due to their limited motility, individual plants experience elevated levels of spatiotemporal heterogeneity. When changes are relevant to fitness and conditions are sufficiently predictable, natural selection favors phenotypic plasticity over genetic differentiation.^1–4^ However, phenotypic modifications often require significant time, during which the environment can change before the products of plastic changes are functional, resulting in mismatches between the modified phenotype and the altered environmental conditions.^5–10^ This implies that selection is expected to favor *anticipatory* plastic responses to cues and signals that are tightly correlated with forthcoming conditions.^9–14^ For example, in sun-loving plants, early perception of imminent shade commonly elicits shade responses even before light competition has materialized,^15–17^ or accelerated seed germination, which could furnish a competitive advantage to early germinates.^18–20^ Additional anticipatory responses have been demonstrated to forthcoming competition,^21,22^ drought stress,^23,24^ changing nutrient availability,^14,25,26^ neighbor proximity,^16^ and salinity.^27^ In some cases, information regarding probable impending contingencies is perceived from neighboring plants,^12,28,29^ with the most studied example being the ‘talking trees’ phenomenon: in response to herbivory, some plants not only increase their local and systemic defenses e.g^30–32^ they also release volatile organic compounds (VOCs), which induce defensive priming in their undamaged neighbors reviewed in^12,33^ Some plants release VOCs in response to abiotic stresses such as drought or salt, which in turn elicit stress priming and tolerance in neighboring plants.^34,35^ Interplant communication of stress cues has also been demonstrated between roots. Unstressed *Pisum sativum* plants not only swiftly close their stomata in response to cues emitted by the roots of their drought-stressed or ABA-treated neighbors, but also induce similar responses in additional unstressed plants located further away from the stressed plant.^29,36–38^ Despite acknowledging the importance of the integration of above- and belowground information,^39,40^ these phenomena have typically been investigated separately^39^ but see^41–43^

Interplant communication may be influenced by the genetic identity and population structure of the involved plants.^44,45^ For example, intra- and inter-plant cueing has been demonstrated to curtail competition between different organs of the same plant,^46–50^ clonemates or kin in various life forms.^51–54^ In *Artemisia tridentata*, priming against herbivory via aboveground volatile cueing was more effective amongst closely related than unrelated plants.^55,56^ An increasing number of studies demonstrate the implications of belowground recognition on competitive discrimination e.g.^54,57^ Despite the increasing number of studies on both above- and below-ground interplant communication,^43,58^ studies of the impacts of genetic relatedness on interplant cueing in both above- and belowground are noticeably scarce.

Here, we investigated the possibility that both above- and below-ground interplant cueing affect plant responses to kin and non-kin cueing of salt stress in the Japanese plantain *Plantago asiatica*, which has been reported to exhibit kin recognition.^20^

## Materials and methods

### Plant material

The study was conducted on Japanese plantain (*Plantago asiatica* L.; Plantaginaceae). *P. asiatica* is a rosette-forming hemicryptophyte, and it inhabits sunny or partially shaded disturbed habitats such as roadsides, woodland trails, and wet grasslands.^59^ *P. asiatica* is wind-pollinated and self-compatible with markedly limited pollen dispersal of 0.1–0.4 m. Outcrossing rate of *P. asiatica* has not been determined; however, in other self-compatible *Plantago* species, it ranges from 3 to 14%.^60^ Due to limited seed dispersal, siblings often grow in clumped patches adjacent to their mother plants.^59,61,62^ Seeds of *P. asiatica* were collected from three populations, with decreasing genetic relatedness – Aomori city A (40°58′ N, 140°47′ E), Aomori city B (40°62′ N, 140°34′ E), and Fukuoka city (33° N, 130° E) (Fig. S1). Fukuoka and Aomori are approximately 1300 km apart (Fig. S1), and the populations in Aomori City A and B are located approximately 10 km apart. Genetic distances between the three populations were determined using genome-wide SNPs (Supplemental methods). All populations were genetically diverse, and as expected, while the two Aomori populations were the most similar to each other, they were highly differentiated from the Fukuoka population (distances between Fukuoka and Aomori A, and between Fukuoka and Aomori B were 0.777 and 0.809, respectively, while the *F*_ST_ value between Aomori A and B was 0.317; Fig. S2). In the studied populations, plants are likely subjected to salt stress due to occasional seawater spray and sea breezes (Terashima and Yamawo et al., unpublished data).

Seeds were collected from 20 mother plants in each population, separated by ≥5 m, during September and October 2015, and stored at 4°C. In April 2016, seeds were sown at a depth of 2 cm in 7 cm wide × 9 cm long × 5 cm deep germination flats. At day 20, when plants had 3–4 leaves, seedlings were transplanted into 130 ml 6 cm wide × 6 cm deep, plastic pots filled with commercial garden soil, containing 70% grass charcoal, 20% vermiculite, 10% pumice, including 8% NPK (Sun & Hope Co., Tokyo, Japan). The soils were sterilized by autoclaving at 120°C for 20 minutes before use in the experiment. Plants were grown in a growth chamber at 25*°C*, with a 16/8 light-dark cycle and 100 µmol *m*-2 s-1 of light, for 20 days. They were watered with tap water to field capacity every other day. Salt treatments began on day 30, when the plants had eight leaves and were approximately five cm tall.

### Experimental setup

**Figure 1.**
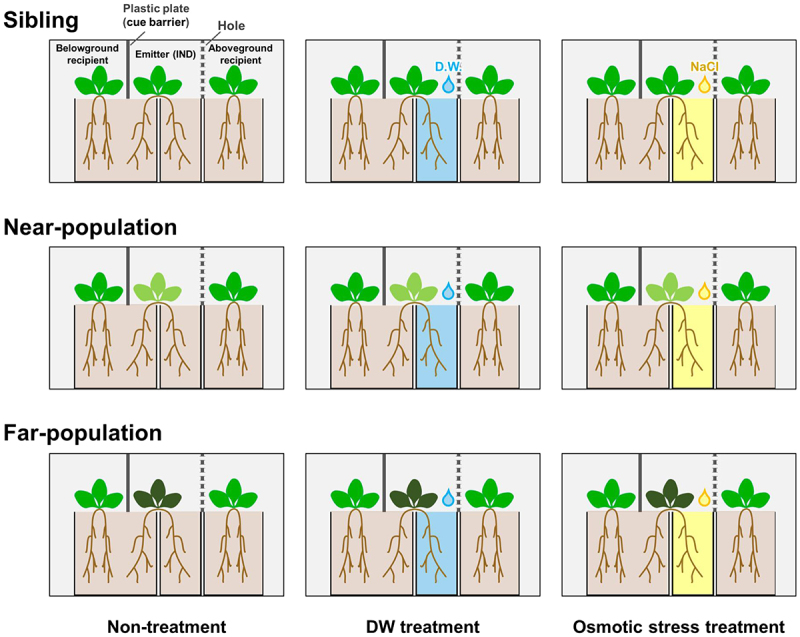
Experimental design to test above- and below-ground communication in three genetic background. Different plant colors indicate different genetic origins. IND, l stress-induced plant. Each experimental condition was replicated 15 times, with five replicates for each genotype.

To separately evaluate via below- and above-ground cueing, we established two types of recipient plants that shared either their rooting or aerial space with a stress-induced (IND) plant (Figure 1). Split-root plants were obtained by equally separating the roots of each plant into two pots (Figure 1). Half of the roots of the central (IND) plant were subjected to salt stress or benign conditions, while the other half shared their rooting volumes with half of the roots of a belowground recipient plant (Figure 1). The proximal parts of the roots and the pot’s rim were coated with a thick layer of petroleum jelly to prevent capillary flow of saline solution between neighboring pots.^29^ The headspace of the IND plants and belowground recipient plants was divided by airtight plexiglass plates. This configuration allowed predominantly interplant root cueing between the IND and belowground recipient plants (Figure 1). In contrast, the aboveground recipient plant did not share roots with the IND plant. It was separated by a perforated plastic plate, allowing only aerial inter-plant cueing between the IND and the aboveground recipient plants (Figure 1). Individual experimental sets were positioned in 36 × 26 × 18 cm glass containers, four m from each other to prevent meaningful volatile cueing between experimental systems.

### Salt treatments and stomatal aperture

Salt treatments started after a 7-d habituation period. In the different treatments, half of the roots of the IND plant (Figure 1) remained untreated (NO), were treated with 15 mL of a 34 mM NaCl solution (SL), or were treated with 15 mL of distilled water (DW).

Stomatal aperture was surveyed as a rapid and highly sensitive indicator of osmotic stress.^38,63^ The number of open and closed stomata was estimated by counting 60 minutes following the onset of the experimental treatments.^37^ The proportion of open stomata was calculated from at least 30 stomata per plant. Stomatal aperture was estimated from epidermal impressions: the lower surfaces of 1 or 2 fully unfurled 20–30 mm^2^ leaflets were molded in a fresh mixture of vinyl polysiloxane dental impression material (Elite HD+, Badia Polesine, Rovigo, Italy). The hardened negative imprints were then copied using clear nail polish, resulting in transparent preparations of positive imprints suitable for microscopic examination at 40x magnification. Because the preparation of the silicone imprints was disruptive, each plant triplet was measured only once.^37^ Stomata were considered open if their width was greater than 2 µm and closed if smaller.

### Genetic background

To estimate the effects of genetic background on interplant cueing, we compared plant responses in sets of siblings and plants belonging to populations from different geographic distances correlated with genetic distance (Figure 1 and S2). We refer to seeds collected from the same mother plant as ‘half-sibling’ (SB), seeds collected from different mother plants from Aomori A or B (Fig. S1 and S2) as ‘near-population’ (NP), and seeds collected from plants at Fukuoka populations as ‘far-population’ (FP) (Fig. S1 and S2). Each of the salt treatments (NO, DW, and SL) was conducted with plants with three genetic backgrounds: SB, NP, and FP (*n* = 5 per genotype), for a total of 15 replications per treatment.

### Statistical analysis

To estimate the effects of genetic background and mode of interplant communication on the effectiveness of interplant stress cueing, treatment effects were compared using a general linear mixed model (GLMM), with a Gamma distribution and a log-link function to accommodate deviations of stomatal aperture data from a normal distribution.^64^ Because the analysis used the Gamma distribution, genotype identity (population) and total number of stomata were included as random effects, and the impact of interplant treatment, genetic background, and their interaction on stomata were tested using the χ^2^-test. Accordingly, the main effects of interplant treatment or genetic background would be significant when their interaction was not significant. Holm – Bonferroni corrections were used where multiple comparisons were conducted on the same data. All analyses were performed using R v. 2.15.1 software.^65^

## Results

**Figure 2.**
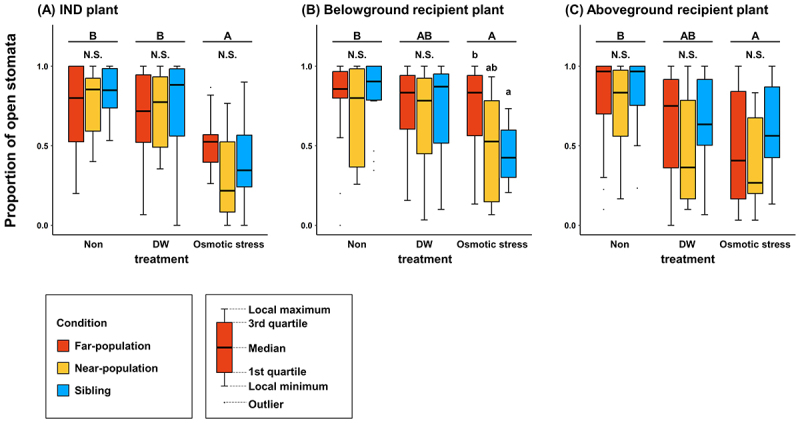
Proportions of open stomata of *plantago asiatica* in three genetic conditions and three stress treatments. The same letter indicates no significant difference (GLMM, *p* < 0.05). Each column had a sample size of *N* = 15.

Under salt stress, IND plants decreased the proportion of open stomata by over 50% compared to DW and Non treatments, regardless of genetic background (Figure 2A). Although the SALT x POPULATION interaction was not significant (*p* = 0.15), the proportion of open stomata of belowground recipient plants was approximately 55% lower in the SB condition compared to control plants (Figure 2B). Under the NP conditions, the proportion of open stomata in belowground recipient plants was approximately 45% lower, with a greater variance and intermediate values, between those of the FP and SB treatments (Figure 2B).

In contrast, in the FP treatment, there was no difference in stomatal aperture between DW and NP. In response to the salt treatment (Figure 2B), the proportion of open stomata was about 30–50% lower in aboveground recipient plants than in their control counterparts, and this response was not significantly affected by the genetic background of the plants (Figure 2C).

## Discussion

Although various studies have demonstrated interplant communication both aboveground e.g.,^12,66^ and belowground,^39–41,67^ these phenomena have been typically investigated separately in shoots and roots^39^ but see^41–43^. Our results demonstrate that in *P. asiatica*, interplant communication of salt stress could involve both above- and below-ground cueing. While aboveground cues conveyed information related to salt stress regardless of genetic background, belowground cueing was more effective between closely related neighbors (Figure 2). These findings are consistent with previous studies with *Artemisia tridentata*, where the effectiveness of aboveground VOC cueing was found to depend on genetic relatedness^55,56,68^ or plant provenance.^33,69,70^ From the selective standpoint of the emitters, reliance on genetic relatedness may increase the inclusive fitness of the communicating plants by directly increasing the survival and performance of other organs of the same plant,^46,49,71^ kin,^47,55,56^ and close relatives.^51,70^ Given that plants have been shown to readily respond to stress cues from unrelated neighbors (Figure 2)^38^ and allospecific neighbors,^37^ a fascinating yet rarely discussed conundrum is why receiving plants should be more attentive to stress cues from their relatives. If and to the extent that plants can recognize the level of relatedness of their neighbors,^72^ it could be hypothesized that closer relatives also possess much more similar adaptations to various stresses. Thus, their cueing is expected to predict imminent challenges more reliably. Extending this rationale, it can be further speculated that plants respond more readily and strongly to stress cues from neighbors belonging to relatively more resistant taxa.^37^ However, these hypotheses should be taken with a grain of salt until they are carefully tested experimentally. Another possibility is that, under natural conditions, both kin selection (private root cueing) and indiscriminate aboveground cueing are utilized under different conditions. Solidago altissima provides a nice example for such a scenario. When protected from insect herbivory, it triggers resistance only in neighbors of the same genotype; however, when subjected to herbivory, it readily elicits anti-herbivory priming in neighboring conspecifics, regardless of their genetic relatedness.^68^

Belowground cueing can involve root exudates, mycorrhizal networks, and root VOCs e.g^29,36,38,39,41,73^ As we used a sterilized soil mixture, the results are likely reflecting the effects of root exudates and/or root VOCs rather than soil microbes. Previous studies have suggested that belowground interplant drought cueing involves abscisic acid (ABA),^29^ a finding also observed in both intra- and interspecific neighbors.^37^ Our results further suggest that interplant cueing of salt stress may involve yet unknown, genetically specific metabolites, calling for further investigation into the identities and modes of operation of these root-exuded cues.

It has been recently suggested that, under some circumstances, belowground interplant cueing could be more effective than above-ground communication.^39^ While aboveground cueing must depend on VOCs, belowground chemical communication may be more protected from wind, extreme temperatures, UV radiation, and oxidation by tropospheric ozone^12,74,75^ In addition, root aggregation in locations of lower mechanical resistance and higher water and nutrient availabilities further increases the likelihood of interplant root encounters and the potential advantage of competitive genetic discrimination.

Taken together, our findings demonstrate the involvement of both above- and below-ground interplant communication in cueing salt stress in *P. asiatica*. Because our study was conducted in highly controlled laboratory settings, further research is needed to determine if and to what extent similar findings can be demonstrated under more natural field conditions. Despite this limitation, we plan to utilize our experimental setup further to investigate the concurrent roles of above- and belowground interplant cueing under variable conditions, such as plant density, resource availability, and stress severity on the relative and interactive roles of above- and below-ground cueing on plant functioning, stress adaptation, reproductive state^28,29^ or genetic relatedness.^20,51^

## Supplementary Material

Supplemental methods.docx

## Data Availability

All datasets are available from the corresponding author on reasonable request.
