# Supplementary material for: Integrated above- and below-ground interplant cueing of salt stress
Source: Plant Signal Behav. 2025 Aug 12;20(1):2542560. doi: 10.1080/15592324.2025.2542560 (PMC12344815; doi:10.1080/15592324.2025.2542560)
Supplement: Supplemental methods.docx [file KPSB_A_2542560_SM0433.docx]

**Supplemental methods**

**Methods in multiplexed inter-simple sequence repeat genotyping (MIG)-seq analysis**

For multiplexed inter-simple sequence repeat genotyping (MIG)-seq analysis, we used eight individuals from each population. Genomic DNA was extracted from silica-dried leaves using the CTAB method (Doyle and Doyle, 1990). A MIG-seq library was prepared using two-step PCR according to the method described by Suyama et al. (2022)Suyama et al. (2022) and sequenced using an MiSeq system (Illumina, San Diego, CA, USA) with an MiSeq Reagent Kit v3 (150 cycles). The raw MIG-seq data were deposited in the DDBJ Sequence Read Archive (DRA, BioProject ID: PRJDB18933).

After removal of primer sequences and low-quality reads using Trimmomatic 0.39 (Bolger et al., 2014), we obtained 4754301 reads (198096 ± 10512 reads per sample) from 5183504 raw reads (215979 ± 11677 reads per sample). We used the Stacks 2.65 pipeline for de novo single nucleotide polymorphism (SNP) discovery (Rochette et al., 2019) with the following parameters: minimum depth of coverage required to create a stack (m) = 3, maximum distance allowed between stacks (M) = 2, and number of mismatches allowed between sample loci while building the catalog (n) = 2. We filtered out SNPs with high heterozygosity (Ho ≥ 0.6), those with fewer than three minor alleles, and retained only those SNPs present in 12 or more samples. Furthermore, PLINK 1.90 (Chang et al., 2015) was used to remove SNPs in linkage disequilibrium with parameter: --indep-pairwise 50 10 0.1. In total, 860 loci and 884 SNPs were used for subsequent analyses. We used SplitsTree4 4.14 (Huson and Bryant, 2006) to construct a Neighbor-Net network based on an uncorrected p distance matrix.

References

Bolger, A.M., Lohse, M., Usadel, B., 2014. Trimmomatic: a flexible trimmer for Illumina sequence data. Bioinformatics 30, 2114-2120. https://doi.org/10.1093/bioinformatics/btu170

Chang, C.C., Chow, C.C., Tellier, L.C., Vattikuti, S., Purcell, S.M., Lee, J.J., 2015. Second-generation PLINK: rising to the challenge of larger and richer datasets. Gigascience 4, 7. https://doi.org/10.1186/s13742-015-0047-8

Doyle, J.J., Doyle, J.L., 1990. Isolation of plant DNA from fresh tissue. Focus 12, 39-40.

Huson, D.H., Bryant, D., 2006. Application of phylogenetic networks in evolutionary studies. Mol. Biol. Evol. 23, 254-267. https://doi.org/10.1093/molbev/msj030

Rochette, N.C., Rivera‐Colón, A.G., Catchen, J.M., 2019. Stacks 2: Analytical methods for paired-end sequencing improve RADseq-based population genomics. Mol. Ecol. 28, 4737-4754. https://doi.org/10.1111/mec.15253

Suyama, Y., Hirota, S.K., Matsuo, A., Tsunamoto, Y., Mitsuyuki, C., Shimura, A., Okano, K., 2022. Complementary combination of multiplex high-throughput DNA sequencing for molecular phylogeny. Ecol. Res. https://doi.org/10.1111/1440-1703.12270
